# Artificial intelligence in health care: laying the Foundation for Responsible, sustainable, and inclusive innovation in low- and middle-income countries

**DOI:** 10.1186/s12992-020-00584-1

**Published:** 2020-06-24

**Authors:** Hassane Alami, Lysanne Rivard, Pascale Lehoux, Steven J. Hoffman, Stéphanie Bernadette Mafalda Cadeddu, Mathilde Savoldelli, Mamane Abdoulaye Samri, Mohamed Ali Ag Ahmed, Richard Fleet, Jean-Paul Fortin

**Affiliations:** 1 Center for Public Health Research, Université de Montréal, Montreal, Quebec Canada; 2 Department of Health Management, Evaluation and Policy, Université de Montréal, Montreal, Quebec Canada; 3 Global Strategy Lab, Dahdaleh Institute for Global Health Research, Faculty of Health and Osgoode Hall Law School, York University, Toronto, Ontario Canada; 4 Department of Global Health & Population, Harvard T.H. Chan School of Public Health, Harvard University, Boston, Massachusetts USA; 5 Research Centre of the University of Montreal Hospital Centre, Université de Montréal, Montreal, Quebec Canada; 6 Faculty of Law, Université de Montréal, Montreal, Quebec Canada; 7 School for Advanced Studies in Public Health, Rennes, France; 8 Research Center on Healthcare and Services in Primary Care, Université Laval, Quebec, Quebec Canada; 9 Research Chair on Chronic Diseases in Primary Care, Université de Sherbrooke, Chicoutimi, Quebec Canada; 10 Department of Family Medicine and Emergency Medicine, Faculty of Medicine, Université Laval, Quebec, Quebec Canada; 11 Research Chair in Emergency Medicine Université Laval-CHAU Hôtel-Dieu de Lévis, Lévis, Quebec Canada; 12 Department of Social and Preventive Medicine, Faculty of Medicine, Université Laval, Quebec, Quebec Canada

**Keywords:** Digital health, Artificial intelligence, Universal health coverage, Low- and middle-income countries, Global health, Public health, Human security, Innovation

## Abstract

The World Health Organization and other institutions are considering Artificial Intelligence (AI) as a technology that can potentially address some health system gaps, especially the reduction of global health inequalities in low- and middle-income countries (LMICs). However, because most AI-based health applications are developed and implemented in high-income countries, their use in LMICs contexts is recent and there is a lack of robust local evaluations to guide decision-making in low-resource settings. After discussing the potential benefits as well as the risks and challenges raised by AI-based health care, we propose five building blocks to guide the development and implementation of more responsible, sustainable, and inclusive AI health care technologies in LMICs.

## Introduction

*“By failing to prepare, you are preparing to fail”*.

(credited to Benjamin Franklin)

In the context of a global epidemiologic transition towards chronic non-communicable diseases (e.g., cancers, cardiovascular diseases, diabetes) [1, 2], approximately half of the world’s population lacks access to basic health care and roughly 100 million people are impoverished as a result of health care spending [3, 4]. In addition, by 2035, the World Health Organization (WHO) anticipates a shortage of nearly 12.9 million health care workers worldwide [5]. To address these challenges, the WHO highlighted in its various drafts and reports the importance of digital technologies to help increase universal access to affordable person- and community-centred care and services [6, 7].

In this regard, artificial intelligence (AI) is seen as a technology that can potentially contribute to the reduction of global health inequalities [6, 8]. AI is defined as “the imitation of human cognition by computers: reasoning, learning, adaptation, self-correction, sensory understanding, and interaction” [9, 10]. Given that most AI-based health applications are developed by, and implemented in high-income countries, their use in low- and middle-income countries (LMICs) is very recent [8]. However, the significant need for health care services in countries with limited resources along with recent developments in the AI field point toward rapid upcoming changes [6, 8, 11]. For instance, while Africa bears 25% of the global burden of disease and is home to only 3% of the world’s health care workers [12, 13], more than 700 million smartphone connections are expected in this continent during the year 2020 [14].

In this paper, we share critical observations and reflections based on our various roles and experience as researchers and experts in digital health and AI, global health, health services and policy research, medicine, public health, health technology assessment, and responsible innovation in health. Some of the authors worked in the field of international development, collaborated with international organizations (e.g., WHO) and/or were born and trained in a LMIC (Mali, Morocco, Niger). The paper adopts a systemic critical perspective with the main objective of stimulating and encouraging debate and further research that address AI as an object of transformation in LMICs’ health systems, not simply as a discrete technical device that can be applied to solve one health problem at the time.

In the following sections, this article discusses both the potential benefits of AI-based health applications in LMICs as well as the risks and challenges that need to be considered and further explored to make way for a more responsible, sustainable, and inclusive use of AI. Readers should keep in mind that several variations are at play both within and across LMICs. While we cannot do justice to these variations, we provide an overview of generic issues that are likely to prove more or less salient in several countries, depending on contextual factors affecting population health needs, human resources, infrastructures, urban/rural divide, etc.

## Potential benefits of AI-based health applications in LMICs

Proponents of the development and implementation of AI-based health applications in LMICs list many potential benefits and advantages, mainly to improve the performance of health systems while reducing costs [4, 15]. For example, such applications could potentially reduce the costs of screening and treatment plan selection for pathologies requiring expensive equipment and specialized expertise unavailable in most hospitals in LMICs, particularly in rural and isolated areas [8, 15–19]. Indeed, when available in local settings, new digital technologies, including AI, can facilitate the development of affordable, better quality, and accessible innovations, while overcoming the local resource-constrained environment [20]. These technologies could counterbalance the necessity for hardware and associated high investments, by leveraging software platforms (e.g., digital apps can “measure body temperature or eye deficiencies, instead of using thermometers or expensive eye measurement apparatus”) [21].

Furthermore, through interactive and private communication, the use of AI chatbots or virtual avatars and characters (e.g., photorealistic virtual representations of clinicians) could help populations suffering from stigmatizing pathologies (e.g., HIV/AIDS, psychiatric pathologies) access care and follow-up services in a timely manner (e.g., advice, recommendations, referrals) [22, 23]. By adapting to local cultures and languages, AI automated translation solutions could also improve access to and use of services, as well as compliance with treatments, in areas where culture or language represent barriers to health care [24].

From an epidemiological perspective, AI could also help predict the spread of pathologies or vulnerability within certain groups or communities, and thus allow for more effective interventions [1, 8, 11]. For example, weather conditions and land use patterns associated with dengue transmission can be identified, while social networks can be exploited to detect infectious disease outbreaks. Finally, AI offers significant potential for maternal and child health, which is one of the major public health issues in LMICs (e.g., pregnancy monitoring, prediction of birth asphyxia, mother and/or child malnutrition) [1, 4, 8].

## Potential risks and challenges of AI-based health applications in LMICs

Because AI-based health applications are recent in LMICs, there are few robust and contextualized evaluations that can guide informed decision-making in these contexts [1, 8]. As such, there is a significant risk of unintentional adverse consequences [25]. Though their use in health care services remains poorly documented, we can identify several major risks and challenges that are specific to LMICs and that are worth considering carefully.

First, to be effective, the training of AI-based health applications requires large amounts of high-quality data and such data is currently unavailable or very difficult to collect in LMICs [4, 8]. Consequently, there is an important risk of developing biased (e.g., gender, ethnicity, age) or defective (“garbage in, garbage out”) AI solutions or of using AI solutions that were trained in contexts that largely differ from the local population [1, 4]. Because the majority of AI-based health applications are trained using data from high-income countries, they may unknowingly be prejudicial or discriminatory towards LMICs populations [11]. Although algorithms are thought to be objective, Faraj et al. (2018) underscore that algorithms are “political by design” in that they are imbued with the values, choices, beliefs, and norms of their developers and of those who assemble the datasets [26]. As such, technology is a “political artifact” with political and social consequences [26]. For example, an AI solution trained with data biased towards over-diagnosis of schizophrenia in African-Americans can have detrimental consequences if used in some sub-Saharan African populations as these technologies could make incomplete or erroneous diagnoses [27]. Moreover, a medical error generated by an AI application could affect a large number of people at the same time, whereas, traditionally, the error of a clinician affects only a smaller number of persons.

Second, one may wonder how the expertise or infrastructures needed to create appropriate governance models will be developed in LMICs in order to guide the use of AI health care technologies [11], which may negatively affect data management, quality and safety of the technologies, and the overall functioning of fragile health systems. The collection, use, storage, and sharing of both individual and population-based data raise important questions in terms of consent, ownership, and access [28]. If not properly governed and managed, there is a risk this data could be used to persecute or marginalize particular individuals, groups, or communities in relation to, for instance, gender, ethnicity, socio-economic group, pathology, or sexual orientation. While some AI technologies can induce ethnicity, which is relevant in certain clinical cases [29], this function could also be used for racial profiling or discrimination [30]. This is particularly relevant as communities in developing countries are highly heterogenous even within a country’s region [31]. Thus, reflexivity by AI developers regarding biases and privacy, and security in data stewardship are vital [8].

In terms of quality and safety of AI-based health applications, the lack of governance may enable companies to commercialize solutions in LMICs that would not obtain regulatory approval in high-income countries. Some could argue that because access to health care can be difficult in certain LMICs, quality and safety requirements should not create obstacles, which may justify a new kind of “medicine for the poor” [32, 33]. Subsequently, a “it’s good enough for them” logic could be the source of new large-scale public health problems [34, 35]. It should be recalled that 70 to 90% of the medical equipment donated to LMICs fail or do not work as expected because of breakdowns (e.g., broken fuses, discharged batteries, lack of spare parts), the lack of user manuals, or the lack of appropriate training for local staff [36–38]. In this regard, the question of who will maintain and update AI-based health applications and with what resources becomes crucial, especially if there is a policy gap [1, 8]. In view of how large expenses and investments in AI can be, some countries may not be able to adopt these technologies beyond the pilot phase. Furthermore, because informal medical care is widely practiced in some LMICs, non-compliant AI applications could spread easily. Since part of the population may consider themselves “lucky” or “privileged” to access some form of health services, they may be unable to challenge or express concerns about the quality and safety of the services provided to them.

In addition to the fragility of their health systems, some LMICs face the challenge of having to implement and coordinate health care delivered by, or overseen by, international development agencies and non-governmental organizations (NGOs). These agencies and organizations usually support particular vertical programs, for instance, malaria control, HIV/AIDS, or maternal health. Hence, their propensity to use AI-based health applications in silos and without a comprehensive vision of other health needs risks further fragmenting and disrupting already fragile health systems, especially by paying little attention to other serious and urgent problems [39]. Failure to consider the realities of local health systems could weaken well-functioning professional, organizational, and community dynamics and practices [40]. For example, the use of AI-based health applications could medicalize certain problems that may be more effectively addressed through poverty reduction, health education, promotion and prevention programs. Consequently, there is also the risk that the budget for AI might divert overall health and social budget and resources [41]. An increasing dependence on AI may also lead to an erosion of clinical skills, critical thinking skills, as well as local practice skills, such as community health practices [15, 32]. There is therefore a risk of newly emerging problems resulting from an over reliance upon AI.

Third, although AI diagnostic tools have the potential to reach and screen rural and isolated populations who lack access to medical experts, their use in such contexts can also raise significant challenges in terms of providing adequate follow-up care, especially when individuals lack the means to reach for and obtain specialized care in large urban centers. AI diagnostic tools developed in high-income countries may recommend treatment plans (e.g., medication, surgery) that are not locally accessible or only available at prohibitive costs in other countries [1]. Obtaining a diagnosis without proper follow-up care may negatively affect quality of life, or even lead to stigmatization within families and communities. This runs counter to the “do no harm” and “test and treat” principles, which are the foundations of medical practice [8], unequivocally accentuating the symbolic North-South divide.

Finally, the use of AI-based health applications in LMICs can involve additional risks for vulnerable and marginalized populations. For instance, mental health AI solutions that can detect psychological traits and patterns could also be used for the interrogation of prisoners or members of certain minorities or dissident communities [24]. In addition, in contexts where men exert control over women’s access to and use of information, technology, and care services, receiving a diagnosis delivered by an AI application on a smartphone could endanger a woman’s safety, especially if the aim of the AI application is to deliver a potentially stigmatizing diagnosis through private communication. In this case, the potential benefit of the AI-based tool meant to protect the privacy of vulnerable populations suffering from stigmatizing pathologies may instead be used as a tool to reproduce symbolic and structural violence, and thus raise a question of human right to security.

In summary, despite numerous advantages of their use, the potential risks and challenges of AI-based applications in LMICs indicate that much work remains to be done before health systems, health professionals, and patients may benefit from these technological advances. This is also the case in several so-called “developed” countries. In line with these considerations, we offer, below, reflections around five building blocks that need to be developed to foster a more responsible, sustainable, and inclusive development and use of AI in LMICs.

## Developing responsible, sustainable, and inclusive AI for health care in LMICs

Innovation in LMICs, despite variations within and across national boundaries, faces realities and challenges in terms of infrastructure and capital that differ from those established in high-income countries [42]. The dynamics of innovation systems could not be fully understood and addressed without carefully examining technological appropriation processes which may vary according to the context [42]. This view requires to move away from the historical trend where “accredited” experts dominate the process of building and interpreting technology, leading to “rhetorical closure” [43]. Indeed, the lack of an inclusive dialogue about these innovations limits the possibilities of gaining a detailed understanding of the scientific, technological, social, and cultural issues innovation raises in LMICs and among historically marginalized communities and groups [44]. AI is no exception to this trend, particularly in view of the current Western dominant discourse about its promises.

In this vein, we propose five building blocks to support further research and discussions promoting responsible, sustainable, and inclusive AI in LMICs: the training and retention of local expertise, a robust monitoring system, a systems-based approach to implementation, and responsible local leadership inclusive of all stakeholders.

Because there is currently fierce competition for AI experts worldwide, the training and retention of local AI experts are essential to ensure that the technology not only follows industry norms and standards, but also respects and meets the needs of local contexts and populations. Training in basic AI “language” and culture as they relate to consent, privacy, and responsible use of AI technologies is necessary for all stakeholders: decision-makers, managers, health professionals, citizens/patients, and communities [1, 25]. Such an objective requires international cooperation to share expertise and lend support, which could be coordinated by international, governmental or non-governmental organizations, and agencies. Towards this end, an international platform could invest part of its own resources to offer consulting services and training for local decision-makers and experts to better understand and respect industry norms and standards as well as evaluate AI technologies (e.g., health technology assessment) [1]. Services could also cover the establishment of appropriate governance strategies and the identification of essential areas for investment and interventions in order to avoid investing in “miracle” solutions hyped by the media (e.g., high-end medical equipment without local infrastructure and expertise) [1, 45].

A robust monitoring system, possibly located at the level of international cooperation agencies/structures, where stakeholders can alert cases of malfunction or misuse as well as troubleshoot and share solutions is also necessary. For such monitoring to be effective and constructive, national and international organizations will need the collaboration of leading digital industry players. Indeed, policies are no longer only in the hands of parliaments or international agencies, but also in digital platforms and codes [23].

Because effective and reliable AI health care technologies are not sufficient in and of themselves, their implementation will require a systems-based approach in order to truly benefit health systems, communities, and patients [46, 47]. As such, contextual needs and practices of each country must be taken into account in order to properly implement the technology (e.g., equity focus may differ from one country to another) [48]. Indeed, certain important health needs in LMICs can be better met through social policies rather than advances in medical technologies, for instance, poverty and inequality reduction, gender equality, or education [33]. In this regard, it is relevant to mention that despite considerable progress in medical technologies and interventions, health inequalities have increased in LMICs because of the declining living conditions of poor populations [49]. Thus, AI should also demonstrate a real benefit in comparison to other interventions that are not necessarily technological. For example, in some rural and/or remote areas, the best intervention may involve the implementation of training and retention programs for on-site health care providers.

Finally, responsible local leadership working with all stakeholders in LMICs will be necessary in order to develop robust AI health care technologies adapted to local contexts and beneficial for local populations [8]. In order to identify and understand health priorities and potential solutions, governments, academic institutions, research centers, international agencies, NGOs, industry, and civil society must be involved in the development and implementation of these technologies [4]. Women, minorities, and poor communities must also play a significant role and have a genuine, legitimate seat at the table in order to guarantee that innovation is truly beneficial, while ensuring that biases and structural inequalities are mitigated [8]. This inclusive approach would allow them to “tell their own version of the story”, to develop a counter-narrative of the dominant sociotechnical vision and participate in the process of collective imagination about AI, which implies fostering an “epistemic justice” [50]. Indeed, “[e]quity should not only be a goal but a sociopolitical process of sustainable change” [51]. Then, these projects could be spaces where constructive exchanges and collaboration between all stakeholders can emerge. Empowering local actors and fostering local collaboration between stakeholders is key to the development of responsible, sustainable, and inclusive AI.

## Conclusion

AI-based health applications may offer many opportunities for LMICs where resources and expertise are lacking and could become a lever to provide access to universal, high-quality, and affordable health care for all. However, if the implementation of this powerful technology is not framed within, and as, an integral part of a global sustainable development strategy, AI may exacerbate public health issues in countries already dealing with substantial problems and urgencies. Within this perspective, it would be relevant to pursue reflections and research on AI development and implementation in LMICs in view of the Sustainable Development Goals, especially around SDG 17 “Partnership for the goals” since productive lessons are likely to be learned in settings that share a number of contextual facilitators and obstacles [52, 53].

## Data Availability

Not applicable.

## Abbreviations

AI: Artificial intelligence
LMICs: Low- and middle-income countries
NGOs: Non-governmental organizations
WHO: World Health Organization
